# The Oxford Domed Lateral Unicompartmental Knee Replacement implant: Increasing wall height reduces the risk of bearing dislocation

**DOI:** 10.1177/09544119211048558

**Published:** 2021-10-26

**Authors:** Irene Yang, Jonathan D Gammell, David W Murray, Stephen J Mellon

**Affiliations:** 1 Nuffield Department of Orthopaedics, Rheumatology and Musculoskeletal Sciences, University of Oxford, Oxford, UK; 2 Oxford Robotics Institute, Department of Engineering Sciences, University of Oxford, Oxford, UK

**Keywords:** Unicompartmental Knee Replacement, dislocation analysis tool, mobile bearing dislocation, lateral knee, implant design

## Abstract

Due to lateral ligament laxity, bearing dislocation occurs in 1%–6% of Oxford Domed Lateral replacements. Most dislocations are medial but they do rarely occur anteriorly or posteriorly. The aim was to decrease the risk of dislocation. For a bearing to dislocate the femoral component has to be distracted from the tibial component. A robotic-path-planning-algorithm was used with a computer model of the implant in different configurations to determine the Vertical Distraction needed for Dislocation (VDD). With current components, VDD anteriorly/posteriorly was 5.5 to 6.5 mm and medially was 3.5 to 5.75 mm. A thicker bearing increased VDD medially and decreased VDD anteriorly/posteriorly (0.1 mm/1 mm thickness increase). VDD medially increased with the bearing closer to the tibial wall (0.5 mm/1 mm closer), or by increasing the tibial wall height (1 mm/1 mm height increase). VDD anteriorly/posteriorly was not influenced by bearing position or wall height. To prevent collision between the femoral and tibial components an increase in wall height must be accompanied by a similar increase in minimum bearing thickness. Increasing the wall height and minimum bearing thickness by 2 mm and ensuring the bearing is 4 mm or less from the wall increased the minimum VDD medially to 5.5 mm. The lower VDD medially than anteriorly/posteriorly explains why medial dislocation is more common. If the wall height is increased by 2 mm, the minimum bearing thickness is 5 mm and the surgeon ensured the bearing is 4 mm or less from the wall, the medial dislocation rate should be similar to the anterior/posterior dislocation rate, which should be acceptable.

## Introduction

Isolated lateral compartment osteoarthritis occurs in about 10% of arthritic knees.^1^ These patients can be treated with Unicompartmental Knee Replacement (UKR), which may either have a fixed or mobile bearing. The lateral compartment of the knee is very different from the medial compartment. The lateral tibial plateau has a convex surface and the lateral femoral condyle rolls back in full flexion and articulates on its posterior surface.^2^ To allow this movement the ligaments are lax in flexion.^3^ An advantage of a mobile bearing is that it is possible to have a convex tibial surface as the contact pressures are low, whereas this is probably not possible with a fixed bearing tibial component as the contact pressures would be very high. A disadvantage of a mobile bearing is that with lax ligaments the risk of dislocation will increase. The Oxford mobile bearing Domed Lateral (ODL) UKR has a spherically convex tibial surface, which has been shown to provide greater roll back and more normal kinematics than a flat surface.^4^ It also has lower linear wear than a fixed bearing device. However the bearing dislocation rate has been reported as being between 1% and 6%.^5^

**Figure 1.**
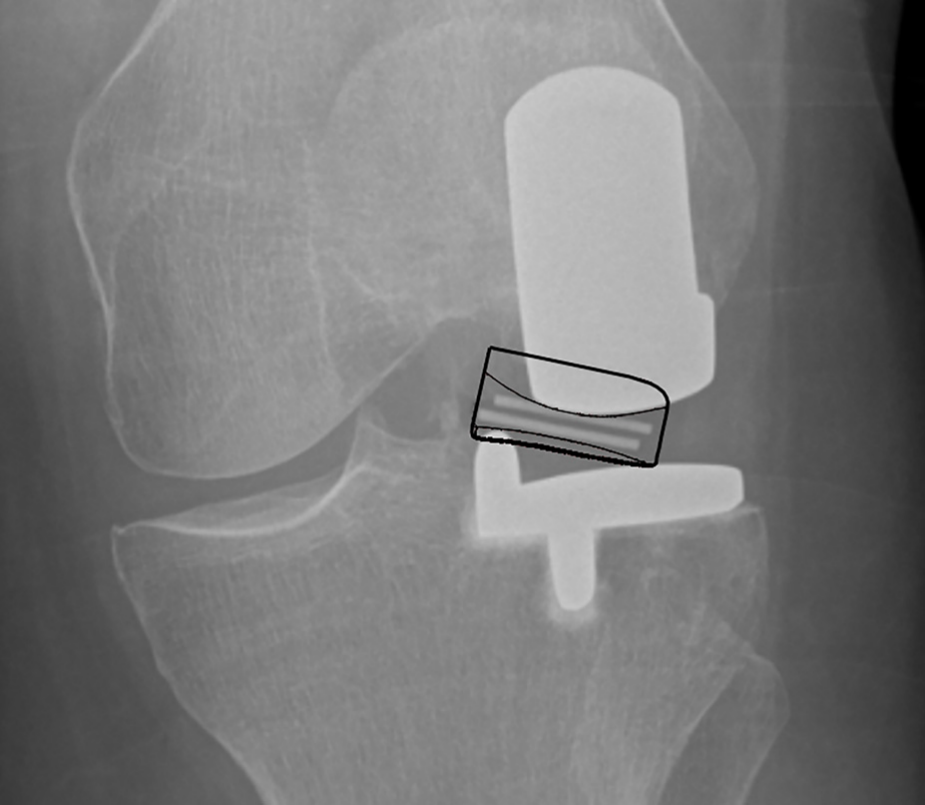
Medial dislocation. The outline of the dislocated bearing can be seen in black and has been positioned using the bearing markers (parallel lines between the femoral and tibial component).

Although the ODL bearing can dislocate anteriorly or posteriorly, the most common type of dislocation is medial,^6^ where the bearing subluxes up onto the wall of the tibial component and becomes entrapped there by the femoral component (Figure 1). The usual treatment for a dislocation is to explore the knee, remove anything such as retained osteophytes that might have displaced the bearing, then insert a new bearing, which may be slightly thicker than the original.^7–9^ In about two thirds of cases there is no further dislocation. Occasionally, if the bearing is very unstable or if there is a recurrent dislocation, surgeons have inserted screws into the tibial eminence to increase the apparent height of the tibial wall.^8,10^ Although this is usually successful in preventing dislocations we are aware of a case in which the femoral component impinged on the screw.

The aim of the surgical technique for the ODL is to accurately restore the anatomy. The spherical surface of the femoral component is positioned where the surface of the femoral condyle was, thus restoring the joint line. The tibial component is positioned so its surface is a few millimetres below the original surface. As the lateral ligaments are tight in extension a bearing of an appropriate thickness that just tightens the ligaments in extension is inserted. If a thicker bearing is inserted the knee will be ‘overstuffed’, which is known to compromise the outcome and increase the bearing dislocation rate.^11^ Therefore, to use thicker bearings the tibia has to be cut lower. The components are implanted approximately parallel with the knee in 90° of flexion. In other positions of the knee, particularly in high flexion, there is rotation in the knee^12^ so part of the femoral component will be above the tibial component. Therefore, if the wall height is increased the two metallic components may collide.^8^ To prevent collision of the metallic components, the tibial cut would have to be made lower and a thicker bearing used. As the knee flexes and extends, the bearing moves backwards and forwards on the tibial component. During these movements there is also rotation within the knee, causing the bearing to move medially in full extension and full flexion. To prevent the bearing from hitting the wall in full extension and full flexion, the components need to be positioned so the bearing is a few millimetres (typically 2–4 mm) from the wall when the knee is in 90° of flexion. The position of the bearing is primarily controlled by the femoral component, which is determined early in the operation. At the trial reduction stage the mediolateral position of the tibial component can be adjusted if the bearing is not ideally positioned.

For a dislocation to occur, the tibial and femoral components have to be distracted, with the amount of distraction required to allow the bearing to dislocate being the ‘entrapment’. The lateral ligaments are lax in flexion so dislocation tends to occur in flexion. The amount of distraction possible will depend on the length and stiffness of the ligaments and the load applied to the knee, which varies considerably between patients. Therefore, the risk of dislocation depends on the entrapment. Based on the concept of bearing entrapment, a validated computer model of mobile bearing dislocation was developed. A detailed description of the development and validation of the tool is published elsewhere.^13^ In brief, the tool allows the femoral component to be incrementally distracted away from the tibial component (mediolaterally and vertically) until dislocation of the mobile bearing was possible.^13^ In the tool, a robotics path planning algorithm explored the possibility for the mobile bearing to manoeuvre its way from a non-dislocated start position to a dislocated goal position without colliding with the femoral and tibial components. Once dislocated, the Vertical Distraction to Dislocation (VDD), which is a measure of bearing entrapment, was recorded. The tool has been validated with data obtained using a custom built mechanical rig, showing 98.7% (95% CI: 0.970–0.994) accuracy for medial dislocation results.

The aim of this study was to use the validated dislocation analysis tool to assess how increasing the bearing thickness, or the tibial component wall height influences medial, anterior and posterior bearing dislocation risk of the ODL replacement.

## Methodology

Computer Aided Design (CAD) 3D models of ODL components were made using Solidworks 2020 (Dassault Systemes SOLIDWORKS Corp., Waltham, MA, USA). The components modelled were a medium size femoral component, size C dome lateral tibial component with normal wall height and wall heights increased by 1, 2, 3 or 4 mm, and medium size dome lateral bearings with nominal thickness of 3, 4, 5, 6 and 7 mm. From the Solidworks part models, stereolithography (STL) files were created. The STL models were inserted into the robotics dislocation analysis tool to computationally assess the risk of Medial and Anterior/Posterior (AP) dislocation.

**Figure 2.**
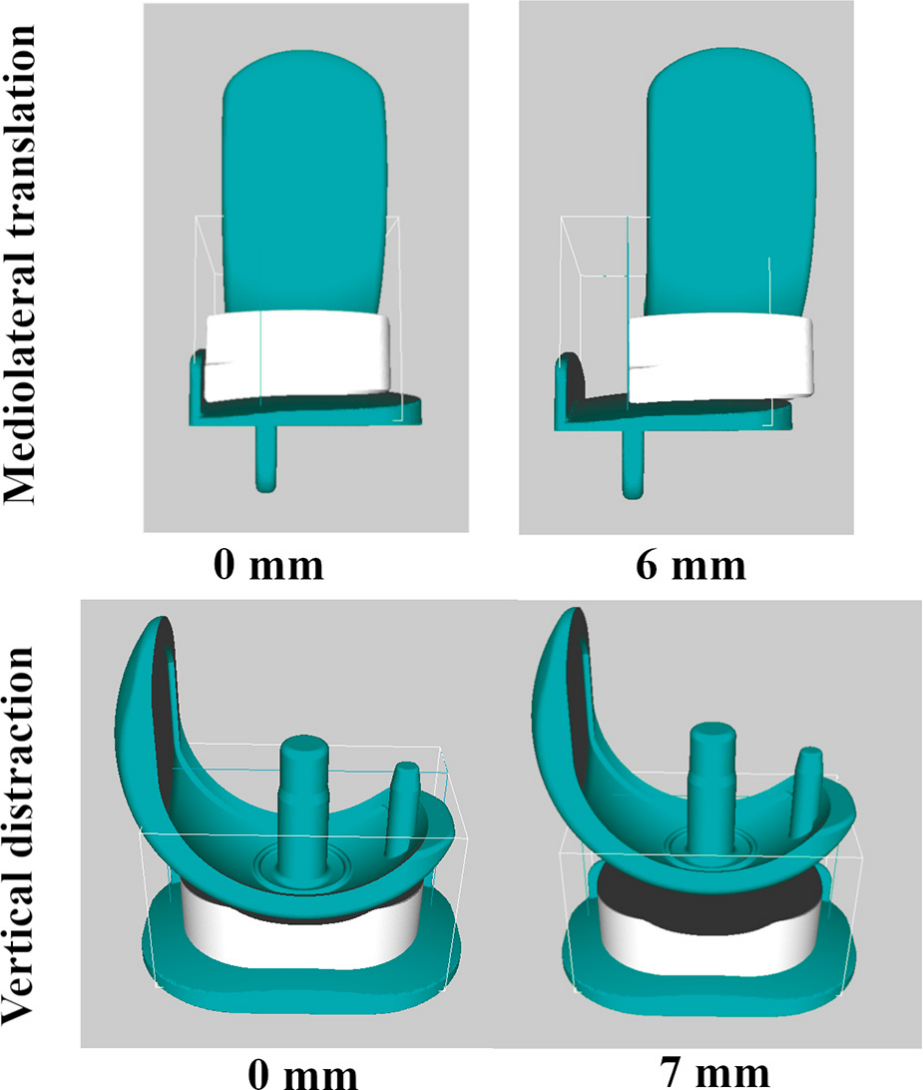
ML translation (0 and 6 mm) and vertical distraction (0 and 10 mm) in the robotics dislocation analysis tool. The teal coloured femoral and tibial components are fixed whereas the dark grey mobile bearing is the robot (unfixed).

For each configuration in the decision analysis tool, the relative position of the femoral component to the tibial component controlled the position of the bearing due to the congruent nature of the components. Therefore, the starting configuration was setup such that the bearing was sitting flush against the tibial component wall (Figure 2). The tibial component was then moved medially in 0.25 mm increments so that the mediolateral (ML) distance between bearing and tibial component wall increased from 0 to 6 mm (Figure 2).

**Figure 3.**
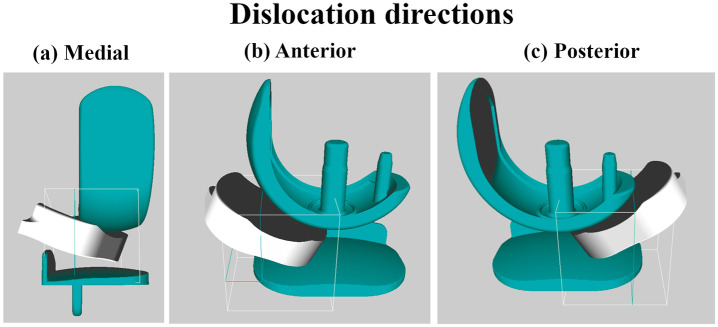
Dislocation directions: medial, anterior and posterior. Boundary box shown in white outline and goal area shown in teal coloured outline.

At each position, the femoral component was then moved vertically away from the tibial component (0–8 mm) in 0.25 mm increments (Figure 2). The dislocation analysis tool used the Rapidly-Exploring Random Trees (RRT) algorithm^14^ to automate the motion of the mobile bearing by randomly sampling incremental steps (1.25 mm) in the space available between the femoral and tibial components such that the bearing can move from the starting non-dislocated position until a dislocated position has been found. The RRT was allowed to run for a maximum of 405 seconds and the search was repeated until a dislocation was found, up to a maximum of 25 searches. A bounding box was used to confine the search region. A medial dislocation (Figure 3) occurred when the centre of the bearing entered the ‘goal area’, defined by the anterior and posterior edges of the tibial component, medially by the tibial wall and laterally by the halfway distance between the midpoint of the tibial component and the tibial wall. An antero/posterior dislocation (Figure 3) occurred when the bearing entered the ‘goal area’, defined medially by the tibial wall, laterally by the lateral edge of the tibial component and the region 10 mm beyond the anterior/posterior edges for antero/posterior dislocation, respectively. When a dislocation was detected, the VDD was recorded (the amount the femoral component had been moved vertically).

In order to determine the minimum distances between the femoral component and the top of the tibial wall, the bearing (irrespective of the thickness) was placed on the tibial component with the bearing flush against the tibial wall. A sphere with the same radius as the femoral component was then placed where the femoral component would sit, in congruent contact with the superior surface of the bearing. For each bearing thickness, the closest 3D distance between the tibial wall and the femoral sphere was identified using the Matlab Euclidean distance measurement.^15^

In this study, we computationally assessed the effect of using thicker bearings on the risk of medial and AP dislocations, starting with the thinnest bearing available which is 3 mm thick. The thickest bearing tested was 7 mm. The current implant design is not at risk of metal-on-metal collision between the metal femoral and tibial components. Therefore, in planning and carrying out testing it was necessary to maintain the existing minimum distance between the metal femoral and tibial components. To maintain the same distance between the femoral component and the top of the wall when the tibial component wall height is increased, we found that the bearing thickness also has to increase by a corresponding amount. E.g. if the tibial component wall height was increased by 1 mm, the thinnest acceptable bearing has to be made 1 mm thicker.^16^ Consequently, we also assessed the effect of different tibial component wall heights, using the corresponding thinnest acceptable bearing, determined by increasing the nominal 3 mm bearing thickness by the same height the wall was increased.

## Results

**Figure 4.**
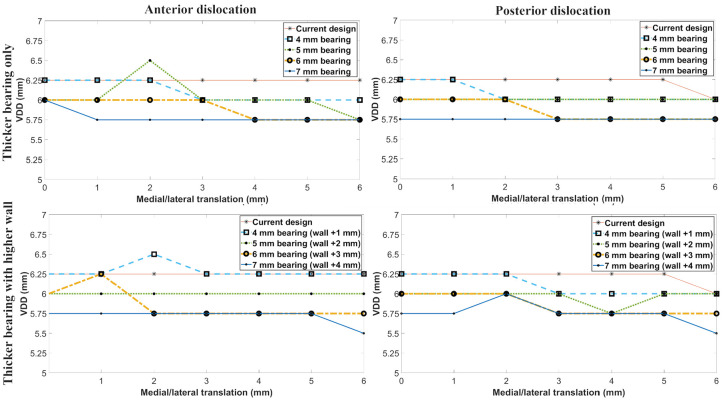
The effect of bearing thickness and higher tibial wall height on anterior and posterior dislocation. Current design refers to the 3 mm bearing with standard tibial component wall height.

The VDD for an anterior or posterior dislocation was identical. It progressively decreased with increasing bearing thickness: with a 3 mm bearing the VDD was 6.25 mm and with a 7 mm bearing, the VDD was 5.75 mm. The VDD anteriorly/posteriorly was independent of wall height: increasing the tibial component wall heights correspondingly with the bearing thicknesses, did not affect the VDD anteriorly/posteriorly (Figure 4).

**Figure 5.**
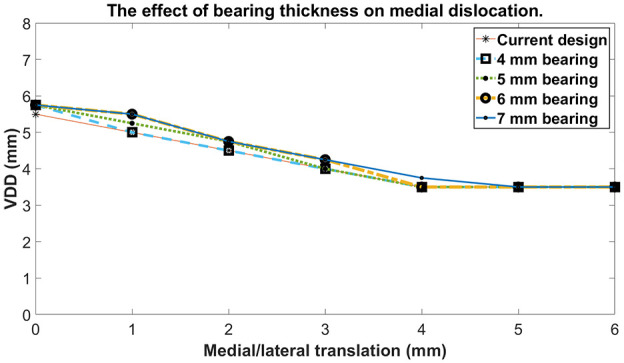
The effect of bearing thickness on medial dislocation. Current design refers to the 3 mm bearing with standard tibial component wall height.

For a medial dislocation, with a 3 mm bearing and the standard wall height, the VDD was 5.5 mm when the bearing was touching the wall (Figure 5). As the femur and bearing were moved laterally the VDD progressively decreased becoming 4.5 mm with the bearing 2 mm from the wall and 3.5 mm with the bearing 4 mm from the wall. Further lateral movement of the femur and bearing (up to 6 mm) did not alter the VDD.

For medial dislocation using thicker bearings increased the VDD a small amount. For example with the bearing positioned flush against the wall, the VDD increased by about 0.25 mm when the bearing thickness increased from 3 to 6 mm. When the bearing was more than 4 mm from the wall bearing thickness had no effect on the VDD medially (Figure 5).

**Figure 6.**
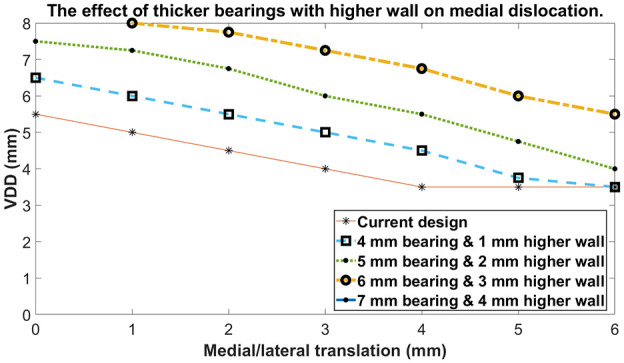
The effect of increasing wall height and using the corresponding thinnest bearing possible on medial dislocation. ‘Current design’ refers to the 3 mm bearing with standard tibial component wall height.

For medial dislocation increasing the height of the wall increased the VDD. For each 1 mm the wall height was increased, the VDD increased by about 1 mm. This increase was independent of the ML position of the femur and bearing and the bearing thickness (Figure 6). The only exception to this was when the bearing was 5 or 6 mm from the tibial component wall, when the increase was smaller. If the wall height was increased by 2 mm then the VDD with bearing flush with the wall, 2 mm from the wall and 4 mm from the wall was 7.5, 6.75 and 5.5 mm, respectively.

**Figure 7.**
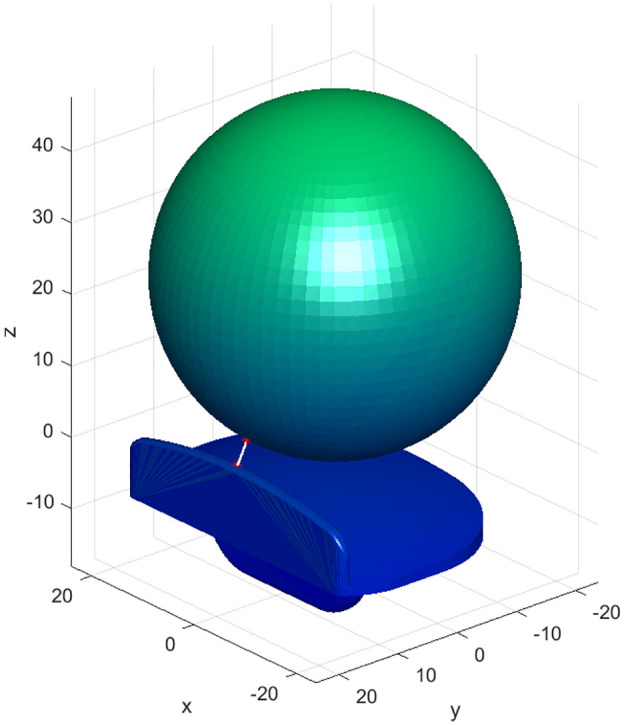
Straight line showing the smallest gap distance between the femoral sphere (representing the femoral component) and the tibial component.

The distance between the surface of the sphere of the femoral component and the top of the wall of the tibial component was related to the height of the wall (Figure 7). The closest distance between the sphere of the femoral component and the top of the standard wall was 1.8 mm and this occurred with the 3 mm bearing and with the bearing touching the wall. With each 1 mm increase in bearing thickness the minimum distance between the sphere and the wall increased by 1 mm. The distance between the sphere and the wall remained at 1.8 mm provided every 1 mm increase in wall height was matched by 1 mm increase in minimum bearing thickness.

As the femoral component is distracted away from the tibial component, the distance between the wall and the femoral sphere increased. For example, with a standard wall and the femoral sphere positioned as it would be with a 3 mm bearing inserted, the closest distance between the tibial wall and the femoral sphere increased from 1.8 to 9.4 mm as the femoral component is distracted vertically away from the tibial component from 0 to 8 mm.

## Discussion

There is considerable variability in the laxity of the ligaments and other soft tissues on the lateral side of the knee and thus the amount the lateral side can be distracted.^17^ Therefore, the risk of a bearing dislocation is related to the entrapment of the bearing which is measured by the amount of vertical distraction required for a dislocation (VDD) to occur using the robotics model. The greater the VDD, the less likely the bearing is to dislocate. For an anterior or posterior bearing dislocation, the VDD was between 5.5 and 6.5 mm under all circumstances. In contrast, for a medial dislocation the VDD was generally substantially less and could be as low as 3.5 mm. It was also influenced by many factors such as the bearing thickness, the ML position of the bearing and the tibial component wall height. The lower VDD medially than anteriorly or posteriorly explains why medial dislocations occur much more frequently than either anterior or posterior dislocations. Anterior and posterior dislocations are rare: occurring in less than 1% of cases.^18,19^ Therefore, if it was possible, by either modifying the implant or the surgical technique, to increase the VDD for a medial dislocation to 5.5 mm, matching the minimum required for an anterior or posterior dislocation, then the overall risk of bearing dislocation would probably be acceptable.

The ML position of the bearing had a marked effect on the VDD medially: the further the bearing is from the wall the lower the VDD and thus the higher the risk of dislocation. The surgeon can control the ML position of the bearing and should make it as close to the wall as possible. During rotation and flexion/extension the lateral bearing tends to move on an approximately circular track, around the medial compartment,^20^ so the bearing is closest to the wall in extension and full flexion. Before making the slot for the keel the surgeon should adjust the ML position of the tibial component so the bearing does not push against the wall in full extension and flexion. This will result in it being about 2 mm or more from the wall at 90° flexion. Given that the position of the vertical saw cut can only easily be adjusted in 2 mm increments the surgeon should be able to position the tibial component so that the bearing is 2 to 4 mm from the wall.

While increasing the bearing thickness only increases the VDD medially by about 0.1/1 mm increase in bearing thickness, clinically, inserting a new bearing, which may be slightly thicker than the original^7–9^ resolves approximately two thirds of medial bearing dislocation cases so that there is no further dislocation. Using a slightly thicker bearing might be resolving clinical dislocations by increasing the tension of the soft tissues in the lateral knee, an thereby reducing the amount that the lateral compartment can distract. However, using a bearing that is too thick can lead to overstuffing the lateral compartment of the knee and overcorrection of limb angle (>7° valgus), overstretching the lateral soft tissues leading to an increased risk of bearing dislocation^11,21^ and progression of disease in the medial compartment of the knee.^22,23^ Therefore, care should be taken to avoid overstuffing the lateral knee.

The factor that has the greatest influence on VDD for medial dislocation is tibial component wall height, with 1 mm increase in wall height increasing VDD by 1 mm. Providing the bearing is 4 mm or less from the wall, an increase in wall height of 2 mm would increase the minimum VDD from 3.5 to 5.5 mm. The minimum VDD for medial dislocation would then be the same as the minimum VDD for an anterior or posterior dislocation so the risk of a medial dislocation should then be similar to that for an anterior or posterior dislocation. As a result the overall dislocation rate should be acceptable.

Although increasing the wall height seems to be a simple way to decrease the risk of dislocation, if the wall height is increased there is a risk that the femoral component might hit the wall, causing damage to the components and leading to metal-on-metal collision with marked metallosis. Currently, at operation the surgeon aims to align the femoral and tibial components so that they are approximately parallel with the knee in flexion. During knee movements such as flexion/extension and rotation, the components rotate relative to each other so part of the femoral component could be above or potentially hit the wall. With the current implant design the minimum distance between the femoral sphere and the top of the wall, which occurs when the thinnest bearing, which is nominally 3 mm, touches the wall, is 1.8 mm. The linear wear rate of the ODL is very low^24^ and we are not aware of any cases where the femoral component has hit the tibial wall, so this distance between the components is safe. To ensure the minimum distance between the femoral and tibial components is maintained using the new implant designs, increasing the tibial wall height by a specified amount would require the nominal 3 mm bearing thickness to also increase by the same height the wall was increased. Therefore, if the wall was made 2 mm higher the minimum thickness of bearing should also be increased by 2 mm from 3 to 5 mm.

If the minimum bearing thickness was 5 mm then to accommodate this, surgeons would have to resect 2 mm more tibia. This would be unlikely to cause any problems. It would require a small amount of the ileo-tibial tract to be released from the upper part of Gerdy’s tubercle, but no ligament release. The situation is very different from that on the medial side of the knee where a deep resection is likely to cause problems as it would require release of part of the deep medial collateral ligament, which might lead to over correction. The use of a 5 mm bearing rather than a 3 mm bearing would have a small additional advantage as it would increase the VDD by about 0.25 mm.

For a dislocation to occur not only does the joint have to be distracted but also the bearing must move or be pushed out of the joint. The main limitation of the study is therefore that only one aspect of dislocation, the distraction, has been studied. However, if the joint has not been distracted enough to allow a dislocation, the bearing would not dislocate. In order to determine if an increased wall height does satisfactorily decrease the risk of dislocation, a cadaver study and then a clinical study will be required.

## Conclusion

The risk of dislocation is related to the VDD measured using the dislocation analysis tool. Anterior and posterior dislocation are rare and have a VDD of 5.5 to 6.5 mm. The VDD for medial dislocation is much lower and can be as low as 3.5 mm, explaining why the risk of medial dislocation is much higher. For medial dislocation VDD decreases markedly with increased wall height (about 1 mm for 1 mm wall height increase), moderately with the bearing being closer to the wall (about 0.5 mm for 1 mm closer) and slightly with thicker bearings (about 0.1 mm for 1 mm thicker bearing). However if the wall height is increased the minimum bearing thickness also has to be increased by the same amount so the femoral and tibial components do not hit. If the wall height was increased by 2 mm, the minimum bearing thickness was 5 mm and surgeons implanted the components so the bearing was 4 mm or less from the wall then the VDD for medial dislocation would be 5.5 mm. The medial dislocation rate should then be similar to the anterior and posterior dislocation rate and it should therefore be acceptable.
